# Polydopamine-Bridged Synthesis of Ternary h-BN@PDA@TiO_2_ as Nanoenhancers for Thermal Conductivity and Flame Retardant of Polyvinyl Alcohol

**DOI:** 10.3389/fchem.2020.587474

**Published:** 2020-09-29

**Authors:** Xiaodong Wang, Weizhao Hu, Yuan Hu

**Affiliations:** ^1^State Key Laboratory of Fire Science, University of Science and Technology of China, Hefei, China; ^2^School of Chemistry and Material Engineering, Chaohu University, Chaohu, China

**Keywords:** hexagonal boron nitride, thermal conductivity, polyvinyl alcohol, flame retardancy, polymer composites

## Abstract

In this study, h-BN@PDA@TiO_2_ hybrid nanoparticles were prepared and used as functional fillers to prepare PVA nanocomposites, and the effects of hybrid particles on PVA thermal conductivity and flame retardant properties were studied. The results showed that hybrid particles could significantly improve the thermal conductivity and flame retardant performance of PVA composites, and effectively inhibit the release of toxic gases such as combustible pyrolysis products and CO, which enhanced the fire safety of PVA composites. When the addition amount of hybrid particles is 5 wt%, the thermal conductivity of PVA composites is 239.1% higher than that of the pure PVA and the corresponding temperature of PVA composites with a mass loss of 5 wt% was 16.2°C higher than that of the pure PVA. This is due to the barrier effect of h-BN and the protective effect of dense carbon layer catalyzed by TiO_2_.

## Introduction

Polymers are widely used in many fields, including aerospace, electronic industry, new energy, insulation materials, decoration, construction, textile, and other fields. However, most polymers do not have flame retardancy, and there is a fire risk during actual use. Fortunately, a large number of studies have proved that adding flame retardants to polymers is an effective way to improve the fire safety of polymeric materials (Shang et al., 2018; Xu et al., 2019; Zhang Q. et al., 2019). Therefore, the performance of flame retardants directly determines the application and development of polymer materials.

With the development of human society, the requirements on material properties are more and more stringent. The concept of “high performance flame retardant” is also increasingly updated, which requires it to have better and more complete performance. The most obvious feature is environmentally friendly, efficient, and multifunctional. It is worth noting that in microelectronics, wearable devices, and other fields, flame retardant, thermal stability and heat conduction properties become the most representative properties of polymer materials used in this field (Cao et al., 2014). However, organic polymeric materials do not have good flame retardancy and thermal conductivity. In order to make the polymer have flame retardancy and thermal conductivity at the same time, it is often necessary to add different functional fillers into the polymer matrix to give the polymer system good flame retardancy and thermal conductivity. Generally speaking, organic polymeric materials do not have good thermal conductivity, so inorganic materials with high thermal conductivity are usually used as fillers to improve the thermal conductivity parameters of polymer system (Safdari and Al-Haik, 2013); compared with organic flame retardants, inorganic flame retardants are relatively green and pollution-free, but in order to obtain better flame retardant effect, the amount of inorganic flame retardant is usually up to 40%. The mixed use of several kinds of inorganic fillers with different functions often leads to the degradation of the mechanical properties of the polymer. Therefore, composite flame retardants with low filling volume are particularly important for the development of polymer materials. The development of nanotechnology has brought solutions to this problem. Therefore, in order to make the polymer have both flame retardancy and high thermal conductivity, multifunctional nano-hybrid flame retardant is the best choice.

The discovery of graphene subverts the understanding of planar two-dimensional (2-D) structural materials. Therefore, the research boom of 2-D layered nanomaterials with similar structures with graphene has been rising in recent years. Some studies have shown that 2-D layered materials (e.g., MoS_2_, GO) can improve the thermal stability and flame retardancy of polymer materials (Huang et al., 2012; Liao et al., 2012; Maddalena et al., 2018; Shi et al., 2019, 2020). The improvement of thermal stability and flame retardant is mainly due to the blocking effect of 2-D layered structure, which can effectively prevent the volatilization of combustible gas and oxygen diffusion, and delay mass loss. As a kind of compound with similar layered structure to graphene, hexagonal boron nitride (h-BN) has good thermal stability and can maintain its layered structure in a relatively stable manner even under thermal conditions; in addition, its special structure can play a barrier effect in the process of polymer combustion (Eichler and Lesniak, 2008; Xu et al., 2013; Weng et al., 2016). Therefore, h-BN can be used as a flame retardant. At the same time, h-BN has a high thermal conductivity, and many researchers use it as a functional filler to improve the thermal conductivity of polymeric materials (Golberg et al., 2010; Feng et al., 2018, 2020; Wang et al., 2018). Therefore, it is very appropriate for our study to use h-BN nanomaterials as functional fillers to enhance the flame retardancy and thermal conductivity of PVA.

As an important metal oxide, titanium dioxide (TiO_2_) has attracted great attention from researchers and materials engineers. It has non-toxic, low price, good light stability, thermal stability, and other excellent performance, widely used in water, air purification, surface self-cleaning, self-sterilization, photoelectric devices, and other fields (Uchida et al., 2002; Mor et al., 2005). Existing research results have shown that metal oxides can promote the formation of char residues during the thermal degradation of polymeric materials (Feng et al., 2016). Continuous dense carbon layer can act as a barrier to improve the flame retardancy of polymer materials and reduce the damage of fire. In recent years, TiO_2_ has also been gradually applied in the flame retardant field.

Zhang Z. et al. (2019) synthesized CeO_2_@TiO_2_ functional hybrid materials by a simple method, and blended them into epoxy resins (EP) as a flame retardant to prepare epoxy nanocomposites. The results showed that the addition of hybrid materials can increase the carbon residual rate of the nanocomposite, reduce the peak heat release rate (PHRR), and total heat release (THR), and reduce the fire risk of polymeric materials. At 700°C, the carbon residues content of the composite can reach about 20%, and the PHRR and THR of the sample decrease to 680 kW/m^2^ and 32.9 MJ/m^2^, respectively. Lam et al. (2011) designed a flame retardant formulation by using nano-TiO_2_, N-hydroxymethyl dimethyl phosphate propionamide, and melamine as the main components, and studied its flame retardant effect on cotton fabrics. It was found that the flame-retardant cotton fabric was extinguished immediately after removing the fire source, and no flame spread. The nano-TiO_2_ composite formula has a significant effect on reducing the flame propagation speed.

In this paper, h-BN nanosheets were obtained by aqueous phase ultrasonic stripping, and then a polydopamine organic layer was obtained on the surface of BN through dopamine self-polymerization, and TiO_2_ was *in situ* grown at the active site provided by the organic layer to prepare core-shell multifunctional hybrid materials (h-BN@PDA@TiO_2_). PVA composites were prepared by using hybrid materials as functional fillers and the effects of hybrid materials on the flame retardancy, thermal stability and thermal conductivity of polymers were studied.

## Materials and Methods

### Materials

Ammonium fluotitanate ((NH_4_)_2_TiF_6_, CP) and boric acid (H_3_BO_3_, ≥99.0%) were purchased from Sinopharm Group (China). h-BN (1–2 μm), PVA (PVA1788, Mw = 80,000, alcoholysis degree: 87.0–89.0%), and dopamine hydrochloride (98%) were purchased from Aladdin (China).

### Preparation of Hybrid Nanoparticles and PVA Nanocomposites

According to literature (Wang et al., 2020), h-BN nanosheets were prepared by liquid phase ultrasonic method. An appropriate amount of h-BN powder was put into a ceramic crucible and calcined at 700°C for 2 h. Then, the powder was cooled to room temperature and washed. The h-BN suspension was ultrasonic treated in the ice bath for 4 h to obtain the h-BN nanosheets.

Preparation of h-BN@PDA: Sufficient Tris-HCl buffer solution was prepared for use. 100 ml fresh buffer solution and 0.4 g h-BN nanosheets were added to a three-necked flask and dispersed by ultrasound for 60 min. Then, 0.203 g dopamine hydrochloride was added into the solution, and the mixing system was magnetically stirred and reacted for 6 h. After the reaction was finished, the product was washed with deionized water until the pH was neutral and collected after drying. And the collected solid powder was the target product of this stage (h-BN@PDA).

**Scheme 1:**
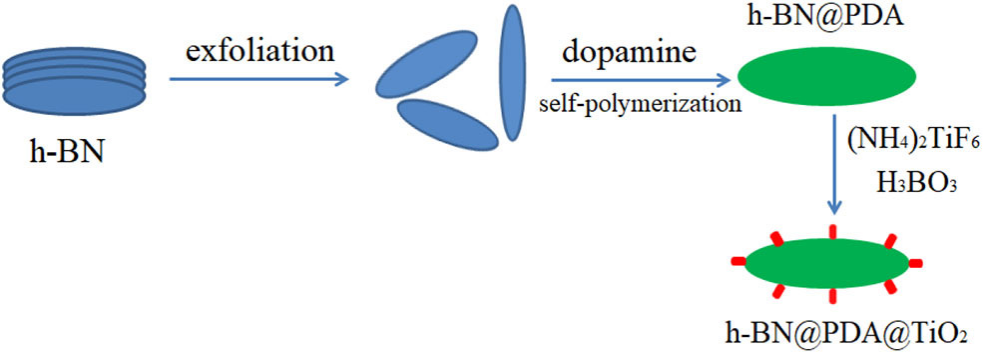
Preparation of h-BN@PDA@TiO_2_.

Preparation of h-BN@PDA@TiO_2_ nano-hybrid materials: 1.98 g of (NH4)_2_TiF_6_ and 1.853 g of H_3_BO_3_ were added to 100 ml deionized water and stirred evenly. Then 0.504 g of h-BN@PDA powder was added into the solution. After the pH value was adjusted to 2.8 by hydrochloric acid, the mixture was poured into a three necked bottle and stirred by magnetic force at 50°C for 12 h. At the end of the reaction, the product was repeatedly washed with deionized water until the pH value was 7, and then dried to obtain h-BN@PDA@TiO_2_ nano-hybrid materials (Scheme 1).

Preparation of PVA composites: Under mechanical agitation, 60 g PVA was added to 300 mL deionized water and heated to 90°C to continue strong stirring until PVA was completely dissolved in the water. Appropriate amount of h-BN@PDA@TiO_2_ nano-hybrid materials was transferred to PVA solution, and stirred at high speed for 15 min, followed by magnetic stirring for 2 h. Then, the mixing system was poured into a mold and naturally dried to form a film.

### Analysis and Testing

Scanning electron microscope (SEM, SUPRA 55, ZEISS), transmission electron microscopy (TEM, TECNAI G20, FEI), fourier infrared spectrometer (FTIR-650, Tianjin Gangdong), X-ray diffractometer (XRD, D/max-2500ps, Regaku), raman spectroscopy (Raman, DXR 3, Thermo Fisher), material testing machine (Model 2663-901/-902, INSTRON), synchronous thermal analyzer (TG-DSC 3+, METTLER TOLEDO), Thermogravimetric-infrared technology combined technology (TG, 209F3, Netzsch; FT-IR, TENSOR27, Bruker), cone calorimeter (CCT, FTT), X ray photoelectron spectrometer (XPS, PHI-5400, PE), thermal conductivity tester (TCi-3-A, SETARAM), energy spectrometer (EDS, xflash 6130, Bruker) were used for analysis and testing.

The combustion test of composite samples was carried out according to ISO 5660 standard procedures, with 100 × 100 × 3 mm^3^ specimens. The thermal conductivity test sample was a circular piece with a diameter of 30 mm and a thickness of 2 mm, and the average value of multiple test data was taken as the result.

## Results and Discussion

### Characterization of h-BN@PDA@TiO_2_

**Figure 1:**
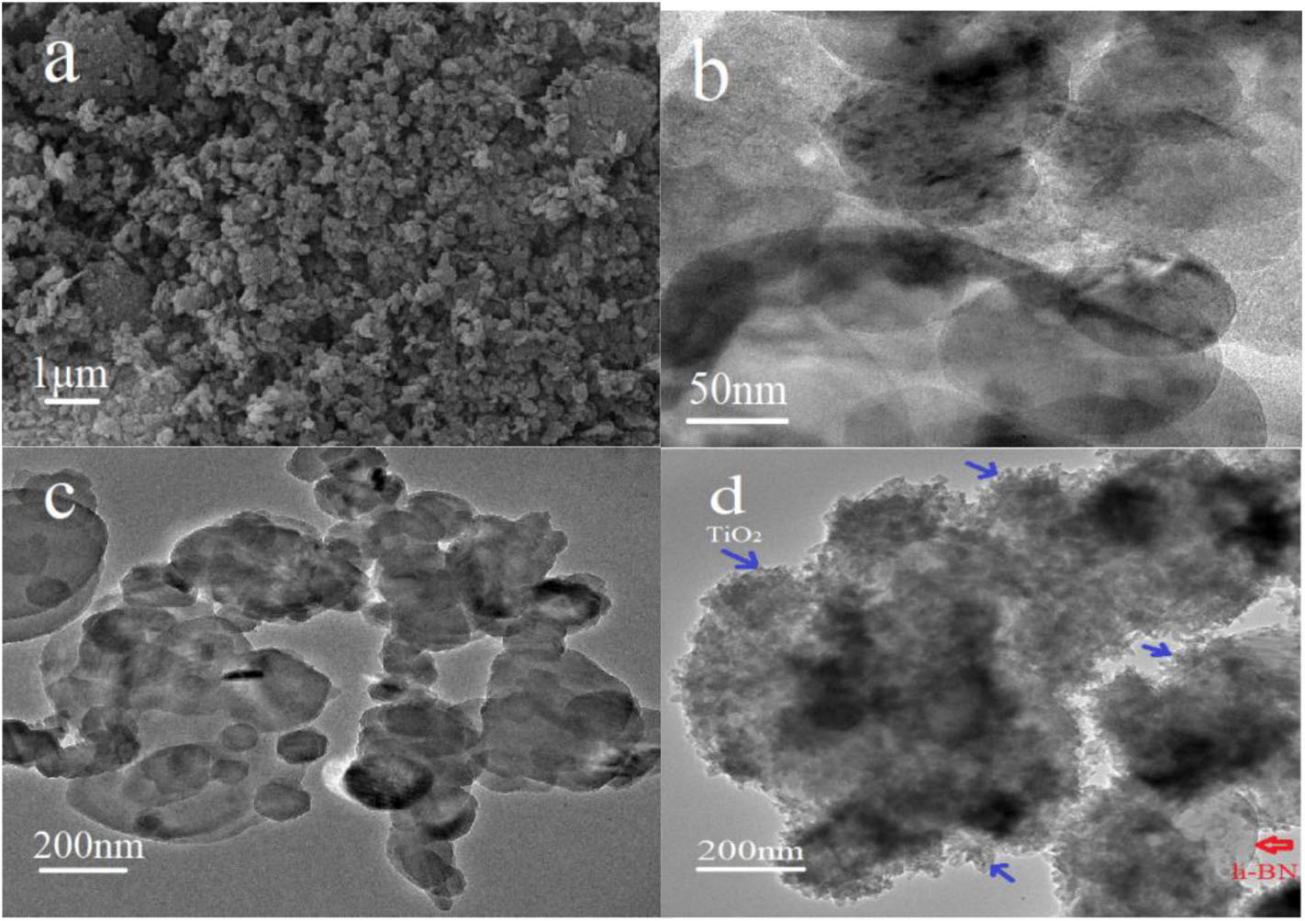
**(a)** SEM image of pristine h-BN; TEM images of **(b)** h-BN nanosheets; **(c)** h-BN@PDA; **(d)** h-BN@PDA@TiO_2_ hybrid particles.

The morphology of pristine h-BN, h-BN nanosheets, h-BN@PDA, and h-BN@PDA@TiO_2_ nano-hybrid particles were analyzed by TEM, as shown in Figure 1. It can be seen from Figure 1a, pristine h-BN particles are thick and compact, and individual particles are flat and blocky. After stripping, h-BN showed obvious thin flake structure with relatively smooth edge and elliptic shape (Figure 1b). Figure 1c shows the TEM images of h-BN nanosheets coated with PDA. The surface of h-BN nanosheets becomes fuzzy. There is a thin, continuous coating. This coating is the product of dopamine self-polymerization, which indicates that the nanosheet has been successfully coated by PDA. And it can be seen from Figure 1d that a number of protuberances are attached to the surface of h-BN nanosheets, which should be TiO_2_ nanoparticles attached by self-assembly on the surface of h-BN@PDA. The h-BN@PDA@TiO_2_ nanohybrid particles have typical core-shell structure, in which the h-BN nanosheet is the core and the PDA organic layer and attached TiO_2_ particles are the shells. And, in the TEM images, no separate TiO_2_ nanoparticles were found except for h-BN@PDA@TiO_2_ particles, indicating that TiO_2_ was not combined with h-BN nanosheets by physical mixing, but was anchored on the surface of h-BN@PDA in the form of *in situ* growth.

**Figure 2:**
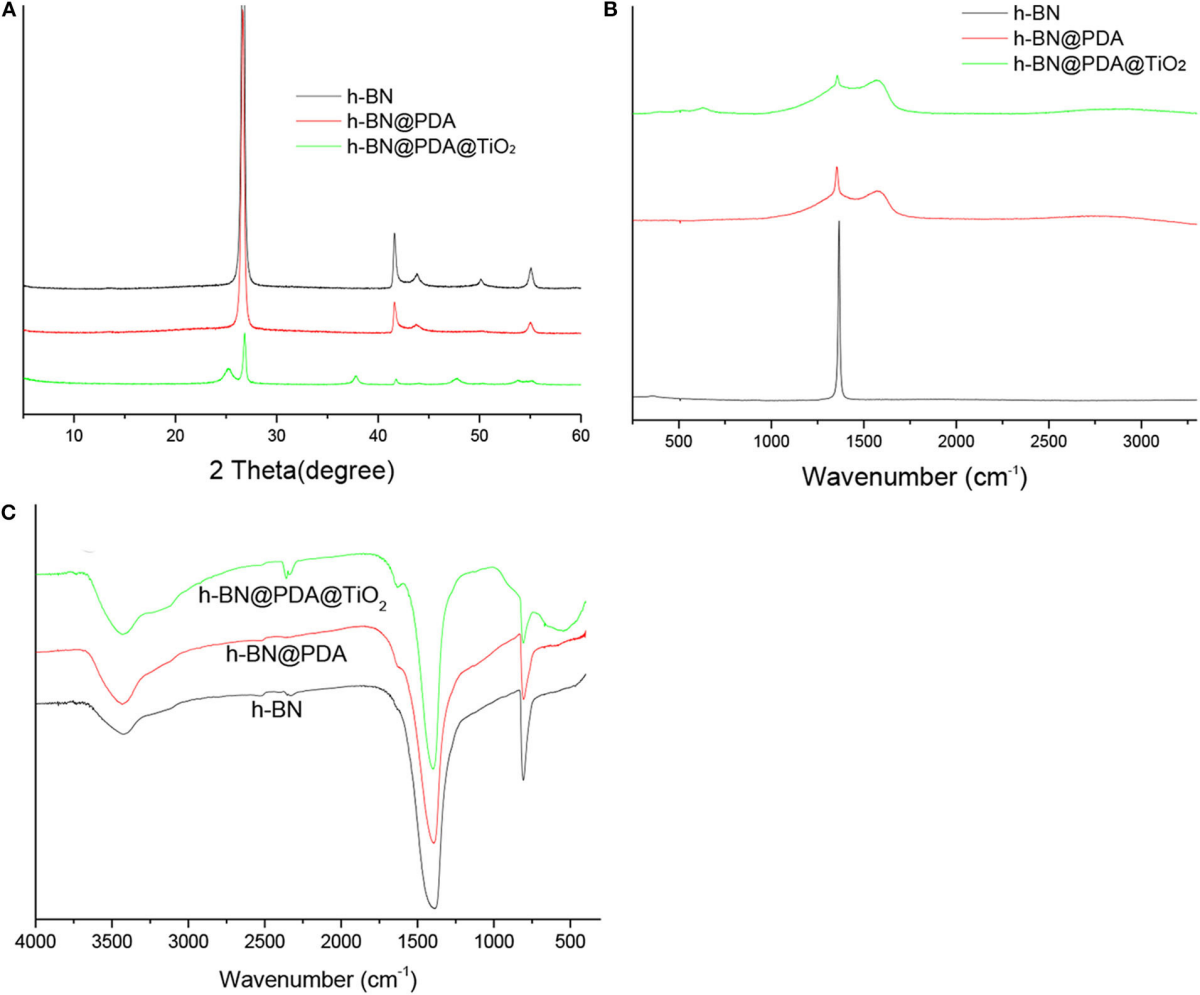
**(A)** XRD patterns; **(B)** Raman spectra; **(C)** FTIR spectrum of h-BN, h-BN@PDA, and h-BN@PDA@TiO_2_ particles.

XRD was used to characterize h-BN nanosheets, h-BN@PDA, and h-BN@PDA@TiO_2_ particles, and the results are shown in Figure 2A. h-BN nanosheets showed obvious diffraction peaks at positions 26.7°, 41.6°, 44.0°, 50.2°, and 55.1°, which correspond to those of JCPDS standard card (NO.34- 042). The difference between h-BN@PDA and h-BN is that there is a PDA organic layer attached to the surface of h-BN nanosheets, which has no influence on the crystal structure of h-BN nanosheets. Therefore, in the XRD spectrum, the diffraction peaks of h-BN@PDA and h-BN are basically consistent. The characteristic peaks of TiO_2_ appeared on h-BN@PDA@TiO_2_ particles, identified as anatase phase of (101) = 25.2°, (004) = 37.9°, and (200) =48.1°, (105) = 53.8° 2-theta values, which also demonstrated that the TiO_2_ particles have successfully grown on the surface of the h-BN nanosheets.

Figure 2B shows the Raman spectra of h-BN, h-BN@PDA, and h-BN@PDA@TiO_2_. The spectra of h-BN, h-BN@PDA and h-BN@PDA@TiO_2_ showed significant differences. Pure h-BN nanosheets have a sharp characteristic peak at 1,366 cm^−1^, which is attributed to the E2g phonon mode (Wu et al., 2004; Gorbachev et al., 2011). After functional decoration, there are two broad peaks at 1,363 and 1,588 cm^−1^ in Raman spectra of h-BN@PDA and h-BN@PDA@TiO_2_ hybrid materials, which are consistent with the reported literature and assigned to catechol tensile vibration and deformation in polydopamine structure (Ku et al., 2010). The results also confirmed the successful conversion of dopamine into polydopamine.

The FTIR spectra of h-BN, h-BN@PDA, and h-BN@PDA@TiO_2_ are shown in Figure 2C. In the FTIR curve of h-BN, the absorption peaks at 1,395 and 804 cm^−1^ correspond to the in-plane stretching vibration peak and out-of-plane bending vibration peak of B-N, respectively. Compared with the FTIR spectra of h-BN, the PDA organic layer on the h-BN nanosheets did not change the FTIR curve significantly. It has been reported that it is difficult to study the surface functional groups of h-BN by FTIR due to the low signal strength of -OH, -NH, and –BN (Zhi et al., 2009). However, when TiO_2_ was attached to the surface of h-BN@PDA, the FTIR curve of the hybrid material showed a wide characteristic peak at 562 cm^−1^, which was attributed to Ti-O vibration peak in TiO_2_.

**Figure 3:**
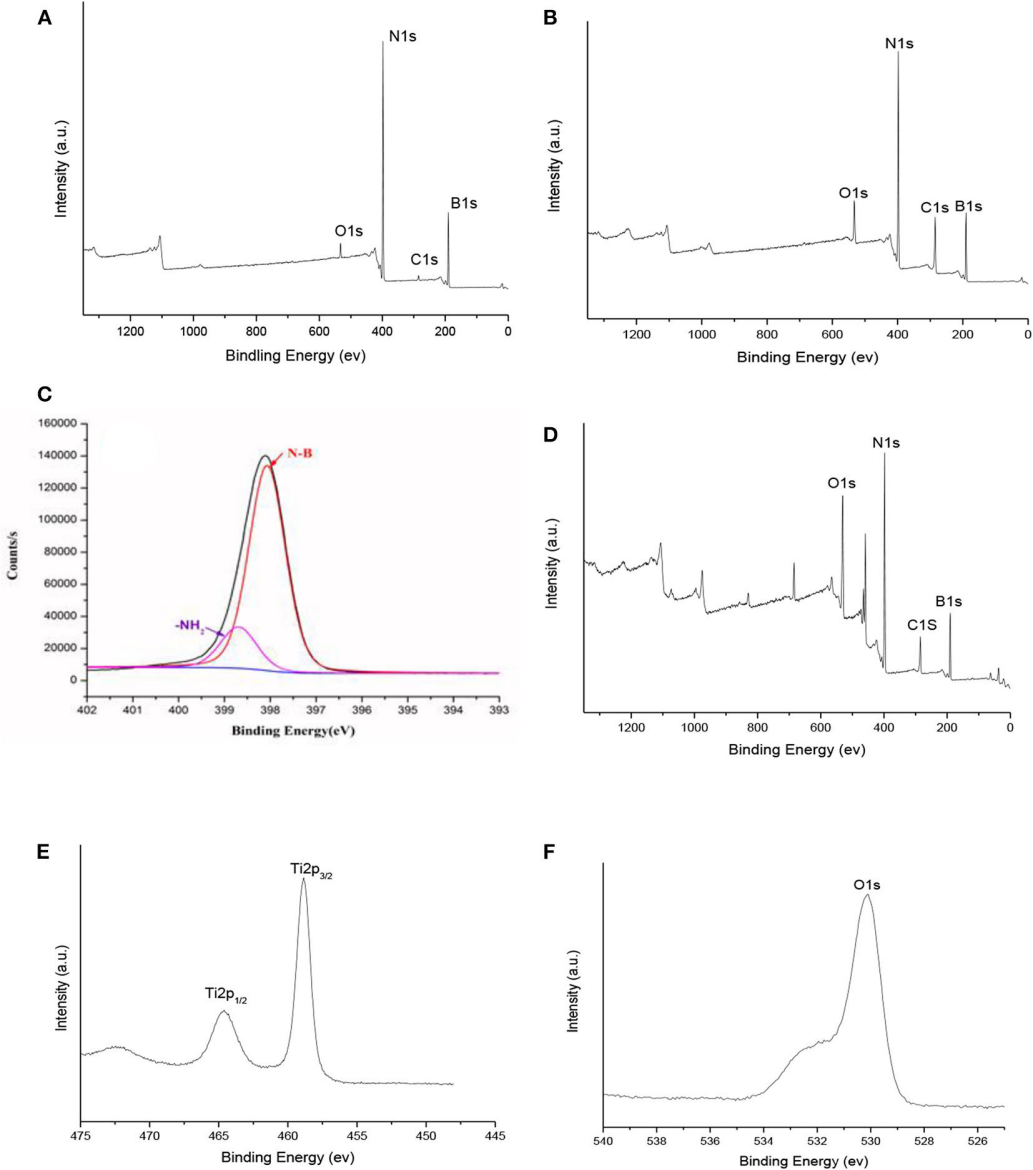
XPS survey scans of **(A)** h-BN; **(B, C)** h-BN@PDA; **(D–F)** h-BN@PDA@TiO_2_ particles.

The surface chemical composition of h-BN, h-BN@PDA and h-BN@PDA@TiO_2_ was further analyzed by means of XPS. Figure 3 respectively show the XPS spectra of pure h-BN, h-BN@PDA, and h-BN@PDA@TiO_2_. According to literature (Cai et al., 2017), the characteristic peaks at 290.1, 193.5, 402.2, and 537.8 ev on the XPS curve belong to C1s, B1s, N1s, and O1s of h-BN, respectively. It is reasonable that C and O elements appear on the surface of h-BN, and the reason may be that the original powder of h-BN contains impurities. As can be seen from the XPS curve of h-BN@PDA, the characteristic peak strength of C 1s and O 1s in the XPS spectrogram of h-BN coated with PDA is significantly enhanced compared with that of pure h-BN. Meanwhile, in the peak separation of N element, the characteristic peak belonging to NH_2_ appears at the position of 399.8 eV. The above results are due to the fact that PDA coating increases the C content on the laminate surface and brings NH_2_ group. The characteristic peaks of Ti and O appeared on the XPS spectra of h-BN@PDA@TiO_2_, which mainly came from the TiO_2_ attached on the surface of h-BN.

### Thermal Conductivity Analysis of Nanocomposites

**Figure 4:**
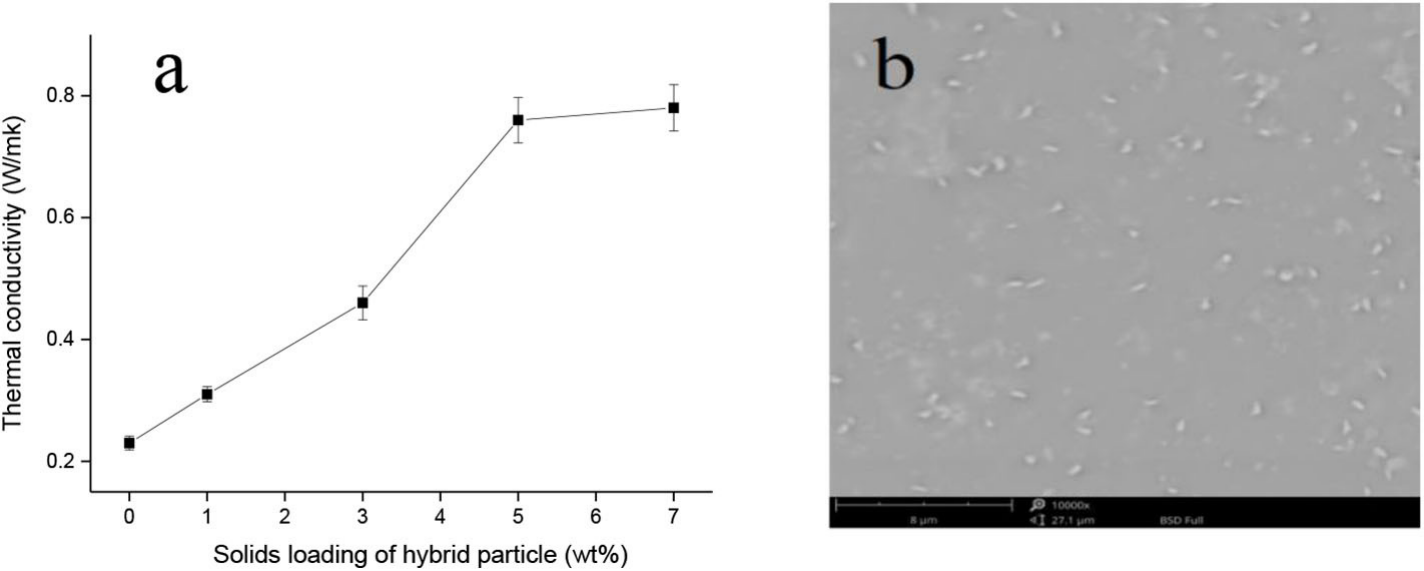
**(a)** Thermal conductivity and **(b)** SEM image of the fractured surface of PVA composites.

Thermal conductivity plays an important role in the long life and high performance of electronic materials. The simplest and most effective way to improve the thermal conductivity of polymeric materials is to introduce inorganic fillers with high thermal conductivity into the polymer matrix. The prepared h-BN@PDA@TiO_2_ hybrid particles were used as fillers to improve the thermal conductivity of PVA. The dispersion of nano-hybrid particles in the PVA matrix can be observed by SEM. As can be seen from Figure 4b, the dispersion of nano-particles in the PVA matrix was relatively uniform. In order to compare the thermal conductivity between PVA and PVA nanocomposites, the test samples of the two systems were prepared under the same conditions. Figure 4a shows the thermal conductivity of PVA and PVA nanocomposites. It can be seen from the figure that the thermal conductivity of pure PVA is 0.23 w·M^−1^k^−1^. When h-BN@PDA@TiO_2_ hybrid nanoparticles were added to PVA, the thermal conductivity of the composite system was significantly higher than that of pure PVA, and increased significantly with the increase of the added amount. When the amount of hybrid particles was increased to 5 wt%, the thermal conductivity of PVA composite reached 0.78w •m^−1^k^−1^, which was 239.1% higher than that of pure PVA. This significant improvement can be attributed to the extremely high thermal conductivity of h-BN nanosheets. As the amount of hybrid particles added in the PVA matrix increased, the thermal conduction network was gradually formed in the polymer system to facilitate the heat transfer. The functional layer coated on the surface of nanoparticles builds a “bridge” in the two-phase interface of h-BN and the polymer matrix, increasing the contact area between the h-BN nanosheets and PVA and improving the two-phase interface characteristics. This improvement is conducive to phonon transfer, thus reducing the interface thermal resistance between h-BN and PVA matrix and promoting the improvement of thermal conductivity of PVA composite system.

### Thermal Stability of PVA Nanocomposites

**Figure 5:**
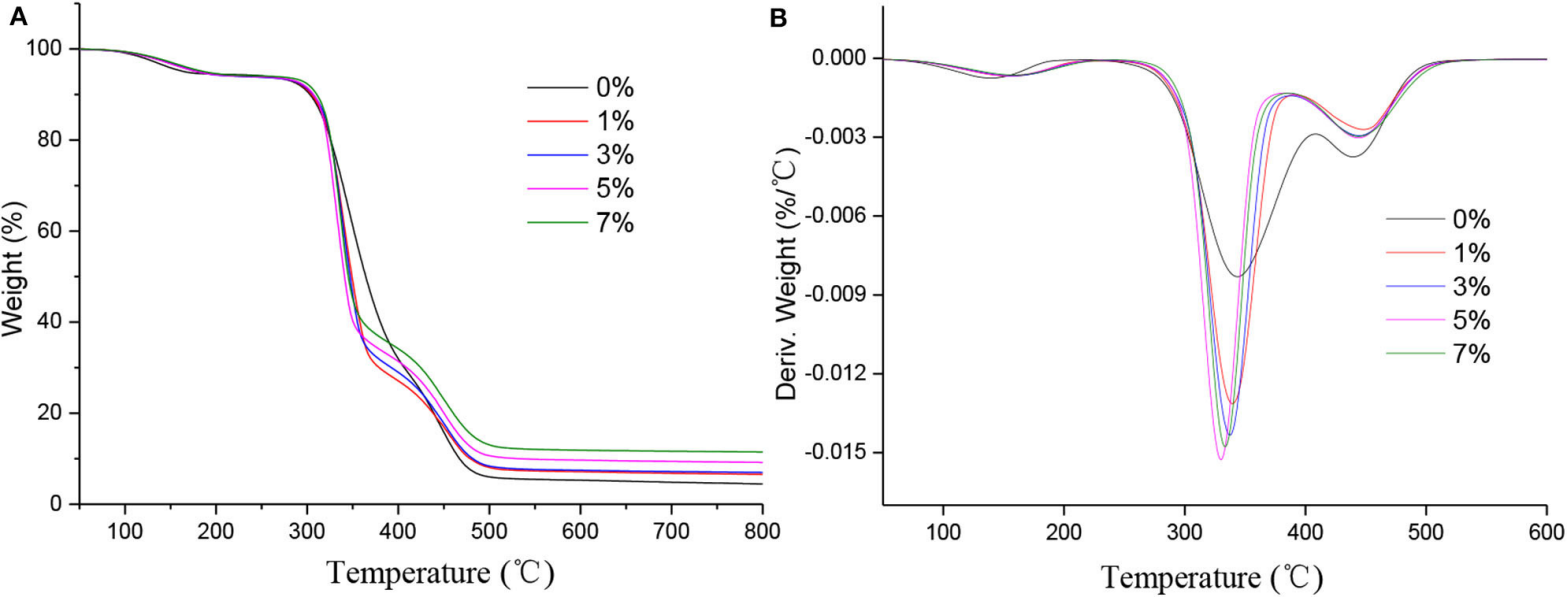
**(A)** TGA and **(B)** DTG curves of PVA nanocomposites.

Thermogravimetric analysis (TGA) is one of the effective methods to analyze the thermal stability of materials. In this study, TGA was used to study the thermal stability of h-BN@PDA@TiO_2_/PVA nanocomposites. Figure 5 shows TGA and DTG curves of PVA nanocomposites, and some important parameters of TGA and DTG curves are summarized in Table 1.

**Table 1:** TGA analysis of h-BN@PDA@TiO_2_/PVA.

**Sample (wt%)**	**T_5%_ (°C)**	**T_50%_ (°C)**	**Carbon residues at 800°C (wt%)**
0	165.5	363.9	4.38
1%	179.8	349.0	6.55
3%	180.2	346.9	6.98
5%	181.7	345.0	9.18
7%	187.3	344.8	11.47

It can be seen from Figure 5 and Table 1 that T_5%_ of PVA composites increases gradually with the increase of the amount of nano-hybrid particles. When the additive amount reached 7 wt%, the T_5%_ of PVA composites reached 187.3°C, which was 21.8°C higher than the pure PVA. This indicates that h-BN@PDA@TiO_2_ has the effect of improving the thermal stability of PVA at low temperature, which is mainly attributed to the thermal stability of h-BN nanosheets itself. However, the T_50%_ of PVA composites was significantly lower than that of pure PVA, which was due to the high thermal conductivity of h-BN and the catalytic action of TiO_2_ which promote the early degradation of PVA at high temperature. This early degradation contributes to the formation of protective carbon layer on the surface of PVA earlier, thus improving the thermal stability of the polymer interior. As the addition amount of h-BN@PDA@TiO_2_ in PVA gradually increased, the residual amount of carbon residue increased from 4.38% of pure PVA to 11.47% of the addition amount of 7 wt%. Based on the cost, the agglomeration of nanoparticles and the influence on the thermal stability of PVA, the appropriate addition amount of nano-hybrid particles as a filler is 5 wt%.

### Flame Retardant Performance Analysis

**Figure 6:**
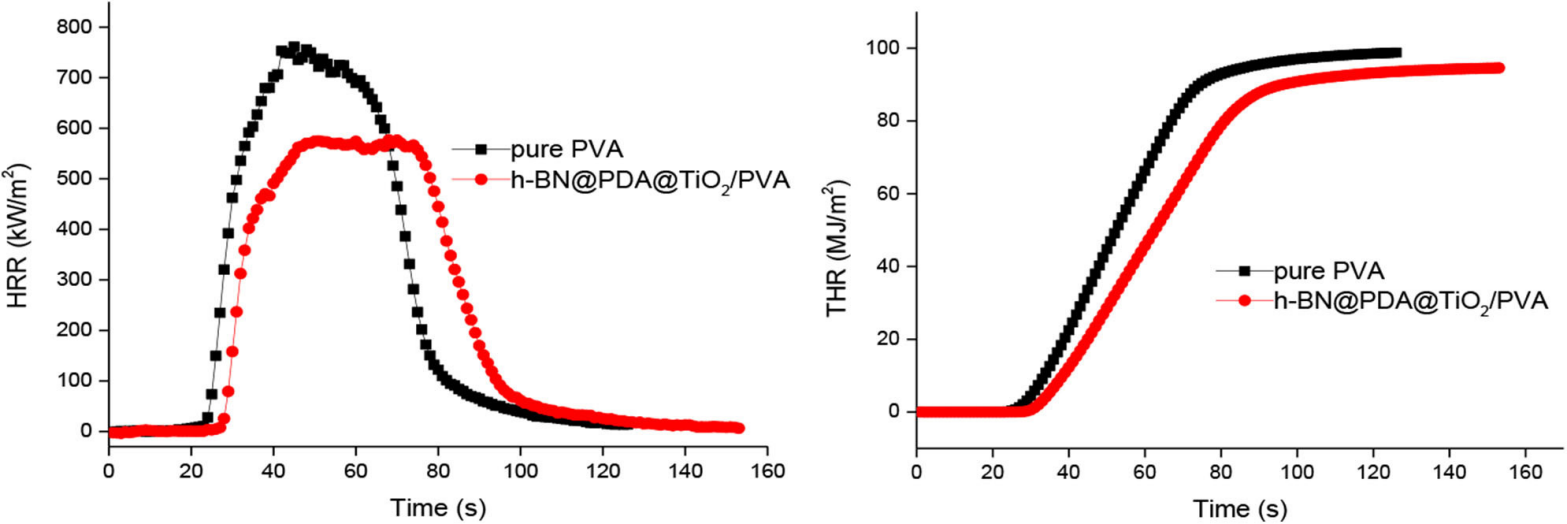
HRR and THR curves of PVA and h-BN@PDA@TiO_2_/PVA composites.

The influence of h-BN@PDA@TiO_2_ on the flame retardant performance of PVA composites can be obtained by cone test, and the results are shown in Figure 6. As can be seen from the figure, the PHRR of pure PVA is 761.39 kW/m^2^, while that of h-BN@PDA@TiO_2_/PVA (addition amount of hybrid particles: 5 wt%) composite is significantly lower than that of pure PVA. The THR of PVA composite was also lower than that of pure PVA. The improvement of the flame retardancy of PVA composite can be attributed to the following two aspects: on the one hand, the “barrier” effect of h-BN 2-D layer structure can inhibit the release of flammable gases during the combustion of PVA; on the other hand, the carbon layer formed by pyrolysis acts as a barrier. TiO_2_ on the surface of hybrid particles can catalyze the formation of carbon, promote the dehydration of PVA into carbon in the combustion process, thus hinder the release of heat and combustible gas and prevent fresh air from entering the combustion area.

**Figure 7:**
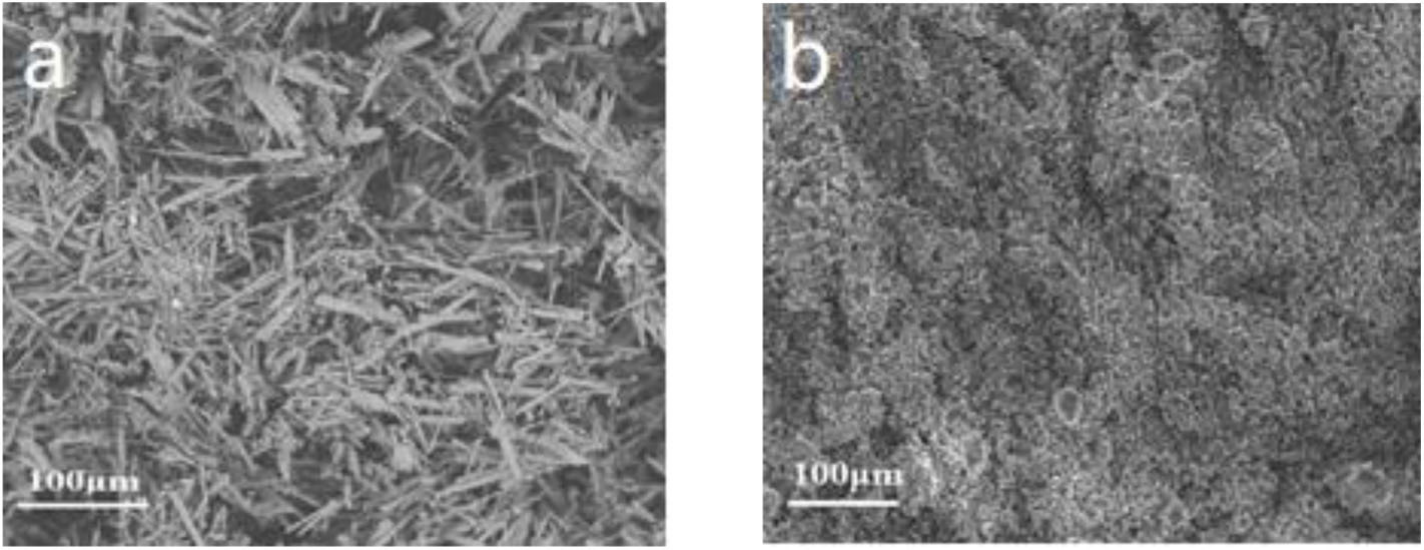
SEM images of carbon residue [**(a)** pure PVA, **(b)** PVA nanocomposite].

SEM was used to analyze the morphology of carbon residue of pure PVA and PVA composite, as shown in Figure 7. It can be seen from Figure 7a that a large number of holes existed in the carbon residue after pyrolysis of pure PVA, and the carbon layer was not compact. The carbon residue formed by PVA composite pyrolysis was compact and continuous (inset in Figure 7b). The reason was that metal oxides in h-BN@PDA@TiO_2_ hybrid particles had a strong catalytic carbonization effect, which promoted the production of more carbon residue in the combustion of PVA composites. This also indicated that the addition of h-BN@PDA@TiO_2_ hybrid particles was conducive to the formation of a compact carbon layer during PVA pyrolysis, which acted as a “protective shell” for PVA to improve the fire safety of PVA.

### Analysis of Gas Phase Products

**Figure 8:**
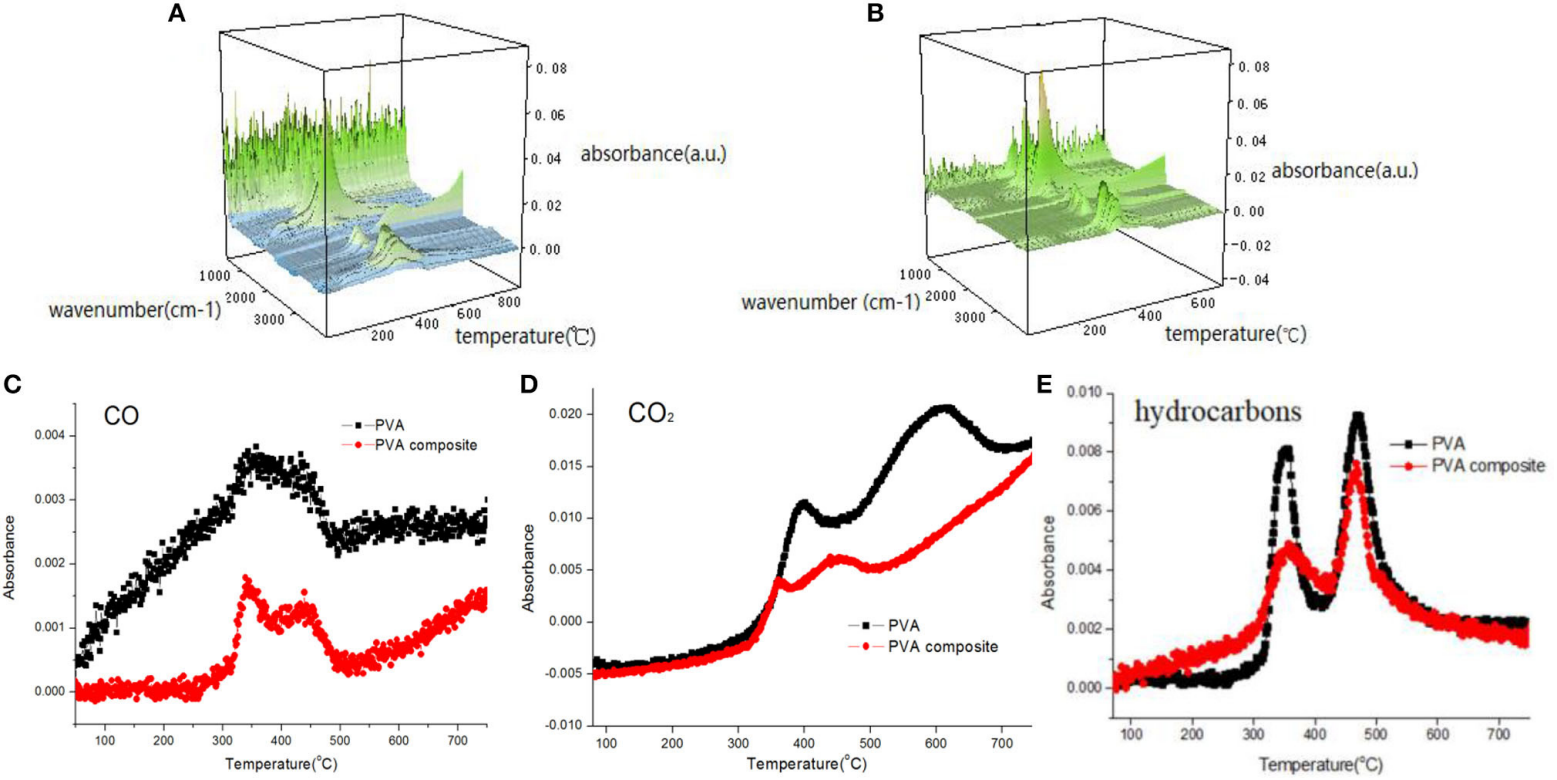
3D-FTIR spectrum of decomposition products [**(A)** PVA, **(B)** h-BN@PDA@TiO_2_/PVA]; absorbance of pyrolysis products for PVA and PVA nanocomposite **(C–E)**.

The gaseous components released during polymer pyrolysis can be tracked and analyzed by means of FT-IR, and then the degradation mechanism of polymer can be studied. The 3D FTIR spectra of PVA and PVA nanocomposites are shown in Figure 8. It can be seen from the Figures 8A,B that PVA composite has similar infrared characteristic peaks with pure PVA. The attribution decomposition products of these peaks mainly include water (about 3,600–3,700 cm^−1^), alkane compounds (about 2,800–3,100 cm^−1^), carbon dioxide (2,300–2,400 cm^−1^), carbon monoxide (2,180 cm^−1^), carbonyl compounds (1,740 cm^−1^) and other organic compounds containing C=C and C-O (1,620 and 1,120 cm^−1^). Figures 8C–E shows the changes of absorbance of pyrolysis products (CO_2_, CO, and hydrocarbons). It can be seen that the absorbance strength of pyrolysis products of PVA composite, including combustible volatiles (hydrocarbons and aromatic compounds) and toxic gas (CO), is lower than that of pure PVA sample, indicating that the introduction of h-BN/PDA/TiO_2_ reduces the harm brought by pyrolysis products. And, the production of hydrocarbons and aromatic compounds is reduced, which avoids the continuous supply of fuel for the combustion zone. At the same time, as a component of smoke particles, the reduction of the production of aromatic compounds reduces the influence of smoke on visibility in the fire field, which is beneficial to the escape of people. The reduction of CO and other gases is of great significance to the life safety of the escape personnel.

### Carbon Residue Analysis

**Figure 9:**
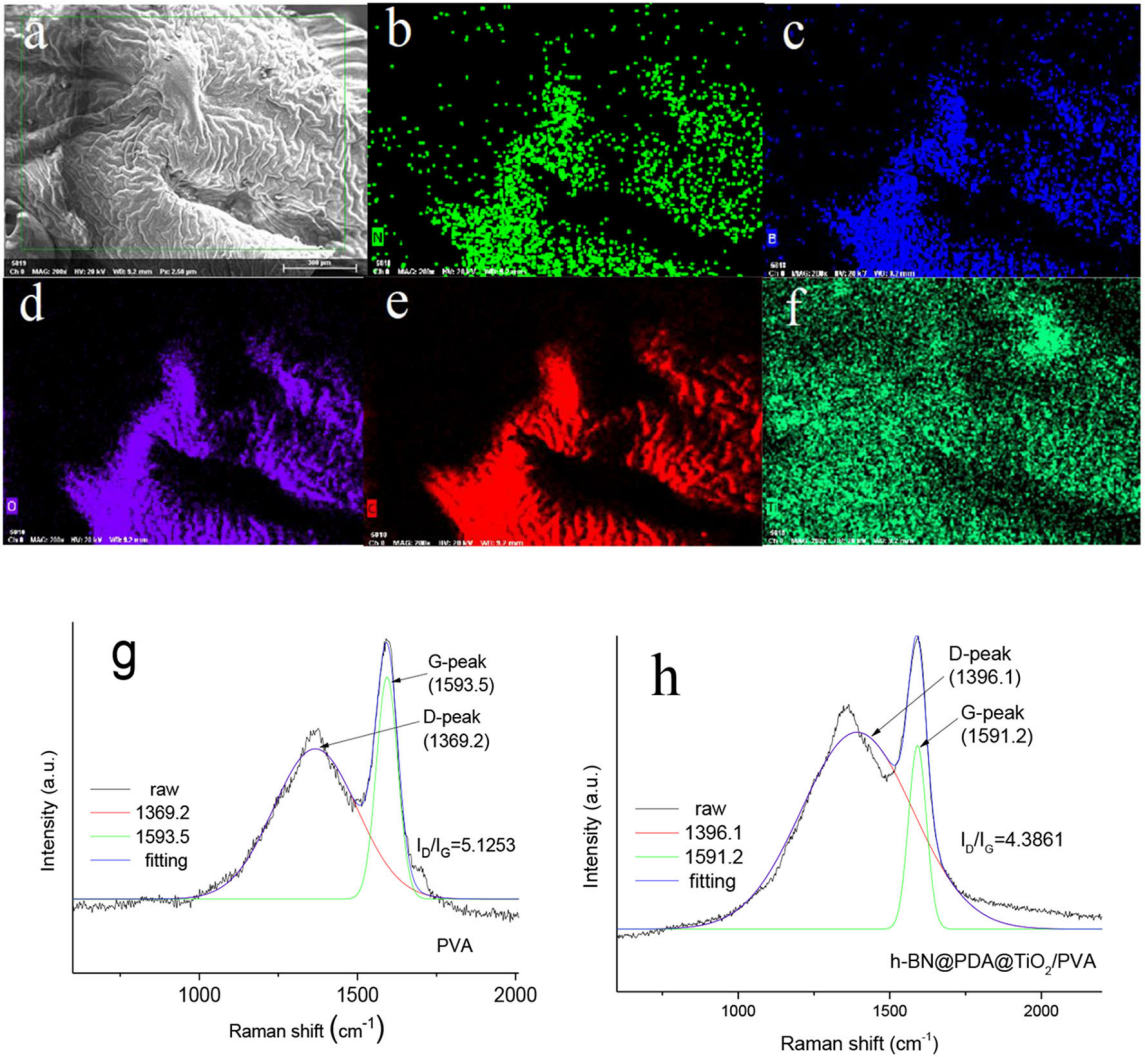
**(a–f)** Element analysis of carbon residue of PVA composite; **(g, h)** Raman spectrum analysis of PVA and PVA composite carbon residue.

The analysis of the chemical composition of carbon residue is helpful to clarify the solid-phase flame retardant mechanism of h-BN@PDA@TiO_2_ during PVA combustion. EDS was used for elemental analysis of carbon residue (inset in Figure 9). As can be seen from the Figures 9a–f that the carbon residue of PVA composite is rich in elements C, O, B, N, and Ti. It can be considered that C and O are mainly from carbonization products of PVA molecules, while elements B, N, and Ti are mainly from hybrid particles. Moreover, it can be clearly seen that Ti is uniformly dispersed in the outer layer of carbon residue, indicating that metal oxides in the system migrate to the combustion zone of the outer surface of the material during the process of PVA pyrolysis, which plays a better role in the pyrolysis of the polymer into carbon.

Figures 9g,h show Raman spectra of carbon residue of PVA and PVA composites. As can be seen from Figures 9g,h Raman spectra of the two samples have similar shapes. And the two peaks of pure PVA carbon residue are 1369.2 and 1,598 cm^−1^, which correspond to D and G bands. D bands represent the symmetric carbon atom vibration of amorphous carbon, while G bands are caused by the 2-D symmetric stretching vibration of SP2 hybrid fossil carbon atoms. After the addition of h-BN@PDA@TiO_2_ hybrid particles, the two peaks of the composite are 1396.1 and 1591.2 cm^−1^. The graphitization degree of carbon layer residue is usually evaluated by the relative aera ratio of D and G bands (I_D_/I_G_) (Tang et al., 2019). Generally speaking, the smaller the ratio, the higher the degree of graphitization. The I_D_/I_G_ value of pure PVA is 5.1253. In comparison, the I_D_/I_G_ value of PVA composites has decreased to some extent. This result strongly proves that h-BN@PDA@TiO_2_ can promote the graphitization of carbon in the carbon residue.

Combined with gas phase analysis and condensate analysis, we can predict the flame retardant mechanism of hybrid fillers. When affected by high temperature heat source, PVA will decompose to produce combustible gas and burn. The heat generated by combustion will accelerate the decomposition of PVA and produce more combustible gas. At the same time, oxygen entering the flame zone will promote the combustion. The combustion of PVA will lose control and bring great fire risk. When the hybrid nanoparticles with 2-D sheet structure are added to PVA, on the one hand, the 2-D sheet structure of h-BN is conducive to prolong the oxygen and heat transfer path, slow down the diffusion of combustible pyrolysis products, and thus inhibit the extension of combustion region; on the other hand, TiO_2_ catalyzes the formation of carbon layer on the surface of PVA., which plays a better protective role on the polymer matrix. The fire safety of PVA is improved by the joint action of the two aspects.

## Conclusion

In this study, h-BN@PDA@TiO_2_ hybrid nanoparticles were prepared and used as functional fillers to prepare PVA nanocomposites, and the effects of hybrid particles on PVA thermal conductivity and flame retardant properties were studied. The results showed that hybrid particles could significantly improve the thermal conductivity of PVA. When the amount of hybrid particles was up to 5 wt%, the thermal conductivity of PVA composite could reach 0.78w • M^−1^K ^−1^, an increase of 239.1% compared with pure PVA. Hybrid particles have an obvious improvement effect on the thermal stability and flame retardant performance of PVA composites, and effectively inhibit the release of toxic gases such as combustible pyrolysis products and CO. As a result, h-BN@PDA@TiO_2_ can enhance the fire safety of PVA composite. This is due to the nano-barrier effect of h-BN and the protective effect of dense carbon layer.

## Data Availability Statement

All datasets generated for this study are included in the article/supplementary material.

## Author Contributions

XW worked on the presented work under the guidance of YH. WH assisted in designing and performing experiments. The manuscript was written by XW and WH. All authors contributed to the article and approved the submitted version.

## Conflict of Interest

The authors declare that the research was conducted in the absence of any commercial or financial relationships that could be construed as a potential conflict of interest. The handling Editor declared a past co-authorship with the authors WH and YH.
